# Ectopic overexpression of mulberry *MnT5H2* enhances melatonin production and salt tolerance in tobacco

**DOI:** 10.3389/fpls.2022.1061141

**Published:** 2022-11-25

**Authors:** Baozhong Zhu, Sha Zheng, Wei Fan, Meirong Zhang, Zhongqiang Xia, Xuefei Chen, Aichun Zhao

**Affiliations:** ^1^ State Key Laboratory of Silkworm Genome Biology, Institute of Sericulture and Systems Biology, Southwest University, Chongqing, China; ^2^ School of Electronic Information and Artificial Intelligence, Shaanxi University of Science & Technology, Xi’an, China

**Keywords:** melatonin, tryptamine-5-hydroxylase, *Morus*, serotonin, NaCl stress

## Abstract

Soil salinization severely inhibits plant growth and has become one of the major limiting factors for global agricultural production. Melatonin (N-acetyl-5-methoxytryptamine) plays an important role in regulating plant growth and development and in responding to abiotic stresses. Tryptamine-5-hydroxylase (T5H) is an enzyme essential for the biosynthesis of melatonin in plants. Previous studies have identified the gene *MnT5H* for melatonin synthesis in mulberry (*Morus notabilis*), but the role of this gene in response to salinity stress in mulberry is remain unclear. In this study, we ectopically overexpressed *MnT5H2* in tobacco (*Nicotiana tabacum* L.) and treated it with NaCl solutions. Compared to wild-type (WT), melatonin content was significantly increased in the overexpression-*MnT5H2* tobacco. Under salt stress, the expression of *NtCAT*, *NtSOD*, and *NtERD10C* and activity of catalase (CAT), peroxidase (POD), and the content of proline (Pro) in the transgenic lines were significantly higher than that in WT. The Malondialdehyde (MDA) content in transgenic tobacco was significantly lower than that of WT. Furthermore, transgenic tobacco seedlings exhibited faster growth in media with NaCl. This study reveals the changes of melatonin and related substance content in *MnT5H2*-overexpressing tobacco ultimately lead to improve the salt tolerance of transgenic tobacco, and also provides a new target gene for breeding plant resistance to salt.

## Introduction

Soil salinization is a growing severe global problem as salinity can impede plant growth and development and reduce crop yields (van Zelm et al., 2020). There is approximately one billion ha of salt-affected soils all over the world (Li et al., 2019). The higher salinity levels have detrimental effects on soil physicochemical and biological characteristics and plant metabolism (Sahab et al., 2021). Salt stress has both osmotic and ionic or ion-toxicity effects on plants (Zhu, 2016). Under salt stress conditions, salt ions accumulate excessively in old leaves through transpiration, causing cytotoxicity and changes in ion homeostasis, resulting in ionic disorders (Debnath et al., 2019; Zhan et al., 2019). Salt stress can also increase the intracellular osmotic pressure and can cause the accumulation of sodium to toxic levels (Zhao et al., 2021). Moreover, salt stress triggers the overdose accumulation of reactive oxygen species (ROS) in crop plants, leading to severe oxidative damage to living tissues (Cheng X. et al., 2021).

Melatonin is an indolic compound derived from tryptophan (Arnao, 2014). It is widespread in animals, plants, and microorganisms. In animals, melatonin plays an essential role in chronobiology, regulating circadian rhythm, improving sleep quality, and positively affecting mood disorders and cancer (Cardinali et al., 2012; Lanfumey et al., 2013; Majidinia et al., 2018; Lok et al., 2019). Phytomelatonin was first discovered in tomatoes and a few other edible crops in 1995 (Dubbels et al., 1995). Research has shown that melatonin is a hormone that acts in many physiological aspects of plants, including seed germination, root organogenesis, flowering, fruit ripening, and stomatal immunity (Park and Back, 2012; Zhang et al., 2015; Yang et al., 2021b; Wang K. et al., 2022). Melatonin also plays an indispensable role in alleviating plants’ bacterial, fungal, and viral diseases (Yang et al., 2021a; Zeng et al., 2022). Moreover, melatonin and its metabolites are powerful antioxidants and free radical scavengers resulting in plant resistance improvement (Sharif et al., 2018; Debnath et al., 2019). These functions improve plant photosynthesis and ionic homeostasis and activate a series of downstream signals, such as hormones, nitric oxide (NO), and polyamine metabolism (Zhan et al., 2019; Niu et al., 2022).

Exogenous melatonin has many positive effects on plants. In the first half of 2022, there were about 250 papers related to the positive effects of exogenous melatonin on plants (www.webofscience.com). However, the use of exogenous melatonin in the horticultural industry continues to face numerous challenges, including identifying effective melatonin levels and developing economically viable melatonin sources (Gao et al., 2022). Previous studies have demonstrated that increasing the endogenous synthesis of the plant is an effective alternative. Overexpression of apple melatonin synthesis gene *MzASMT1* significantly improved drought tolerance of transgenic *Arabidopsis* plants (Zuo et al., 2014). Ectopic overexpression of grape melatonin synthesis gene *VvSNAT1* in *Arabidopsis* resulted in increased melatonin production and salt tolerance (Wu et al., 2021). The number and size of vascular bundles in the culms and leaves were significantly increased in the rice overexpressed melatonin synthesis gene *OsCOMT* (Huangfu et al., 2022).

The classical biosynthesis pathway of melatonin in plants has been elucidated. It begins with tryptophan and consists of four enzymatic steps, and five enzymes are known to be involved. Tryptophan decarboxylase (TDC), Tryptamine-5-hydroxylase (T5H), Serotonin N-acetyltransferase (SNAT), N-acetylserotonin methyltransferase (ASMT), Caffeic acid-O-methyltransferase (COMT). TDC enzymatic conversion of tryptophan to tryptamine. T5H catalyzes the conversion of tryptamine to serotonin. SNAT is the penultimate enzyme that catalyzes serotonin into N-acetylserotonin. COMT and ASMT catalyze N-acetylserotonin to melatonin (Back et al., 2016; Byeon et al., 2016; Zheng et al., 2021b). T5H is the second enzyme in melatonin biosynthesis. It belongs to the cytochrome P450 monooxygenase family, which is a multigene family in plants (Fujiwara et al., 2010; Park et al., 2013a; Park et al., 2013b). The identification of gene *T5H* in mulberry trees has been previously reported and seven *MnT5H*s have been identified in the mulberry genome, with *MnT5H2* at the highest expression (Zheng et al., 2021a).

T5H is an indispensable enzyme in melatonin biosynthesis. However, some studies have reported a negative correlation between the content of serotonin, the catalytic product of T5H, and melatonin content in rice and tomato (González-Gómez et al., 2009; Park et al., 2012; Commisso et al., 2022). And serotonin is also implicated in several physiological roles, such as flowering, morphogenesis, and adaptation to environmental changes (Kang et al., 2007). The mechanism of the T5H effects in plants is intricate, especially in woody plants, with limited reporting.

Mulberry is an economically important food crop for the domesticated silkworm (Jiao et al., 2020). Mulberry is also planted for their fruit, which is often eaten as fresh food or made into juice, jam, and wine because of its tasty and nutritious characteristics (Lee et al., 2013; Shen et al., 2014). Furthermore, mulberry can adapt well to stress, such as metal exposure, high salinity, drought, and flooding (Zeng et al., 2020; Rao et al., 2021). Therefore, mulberry is an important economic forest tree species in many developing countries such as China. In our previous studies, it has been found that mulberry has high efficiency of melatonin synthesis. Amongst the 49 mulberry varieties, the Chuansang mulberry (*Morus notabilis*) has the highest melatonin content. However, the significance of the efficient melatonin synthesis for stress resistance of mulberry has not been analyzed (Zheng et al., 2021a; Zheng et al., 2021b). In order to explore this question, we transformed *MnT5H2*, a crucial gene for melatonin synthesis in mulberry, into tobacco (*Nicotiana tabacum* L.) and examined the changes in the content of each substance in the melatonin synthesis pathway in transgenic tobacco. The ectopic overexpression of *MnT5H2* increases melatonin content in transgenic tobacco. Furthermore, *MnT5H2* enhanced the salt tolerance of transgenic tobacco by improving ROS and ion homeostasis. Overall, our study initially reported the positive regulatory role of mulberry *MnT5H* gene in salt stress and its mechanism, which provides some new knowledges for understanding the significance of the efficient melatonin synthesis for stress resistance of mulberry and a new target for improving plant salt tolerance.

## Materials and methods

### Generation of overexpression-*MnT5H2* tobacco

Based on the gene information of *MnT5H2* (GenBank accession number: Morus018957), *MnT5H2* was amplified by PCR using cDNA from the leaves of *Morus notabilis*. The full-length sequences were cloned into the *pLGNL* overexpression vector. The CaMV35S promoter was employed for control the *pLGNL*-*MnT5H2*. All primers were designed as shown in **Supplementary Table 1**. The *pLGNL*-*MnT5H2* was transformed into *Agrobacterium* tumefaciens strain GV3101. Agrobacterium containing positive plasmid was inoculated into YEB liquid culture medium and incubated to OD_600_ = 0.8, then the bacterium was resuspended with osmotic medium (MS + 10 mM MgCl_2_ + 100 mM AS + 0.01% (W/V) MES) to OD_600_ = 0.8. The resuspended bacterium was transferred into tobacco leaves using the leaf disc transformation of tobacco, and the tobacco leaves were incubated on the co-culture medium (MS + 0.1 mg/L NAA + 0.5 mg/L 6-BA) at 25°C in the dark for two days. After two days, tobacco leaves were transferred to medium (MS + 0.1 mg/L NAA + 0.5 mg/L 6-BA + 100 mg/L Kan + 300 mg/L Cef) and incubated at 25°C with 16 h of light and 8 h of darkness.

### Plant materials and experimental design

Tobacco plantlets were grown in Murashige and Skoog (MS) media (3% sucrose, 0.7% agar, 0.5mg/L IAA, pH 5.7) with a temperature of 26°C, a humidity of 65-75%, and a light duration of 16 h. Then the T0 transgenic tobacco and uniformly grown WT tobacco were transplanted into 7×7 cm^2^ × 8 cm pots for 20 days. For the salt treatment assay, the transgenic and WT tobacco was treated with 10 mL NaCl solution (300 mM) every day until wilting occurred after 10 days. Following this, the third leaf was cut off and preserved in liquid nitrogen for subsequent experiments. To investigate the growth of transgenic tobacco seedlings under salt stress, T1 seeds were germinated and grown in 1/2 Murashige and Skoog (MS) media (3% sucrose, 0.6% agar, 200mM NaCl), and the growth lengths were counted after 12 days. To observe the germination of transgenic tobacco seeds under salt stress, T1 seeds were germinated on filter paper and cotton moistened with salt solution (200mM NaCl), and the germination rate was calculated after 12 days. The NaCl concentration used in the treatment was referred to the previous study (Trombin-Souza et al., 2017; Tok and Temizel, 2021).

### Gene expression analysis

Total RNA was extracted from leaves using the TaKaRa MiniBEST Universal RNA Extraction Kit and used for cDNA synthesis with PrimeScipt™ RT reagent Kit with gDNA Eraser (TaKaRa, Beijing, China) after RNA concentration and quality assay with NanoDrop 2000 (Thermo, shanghai China). The quality was further examined using primers designed with the *NtActin* (U60489). And *NtActin* was used as the internal control in all qRT-PCR analyses. The primer sets used are listed in **Supplementary Table 1**.

### Malondialdehyde, proline, chlorophyll and catalase assays

For physiological index measurements, the content of malondialdehyde (MDA), proline (Pro), Chlorophyll, and the activities of catalase (CAT) were determined with Malondialdehyde (MDA) assay kit, proline assay kit, Chlorophyll assay kit, Catalase (CAT) assay kit, and Peroxidase assay kit (Nanjing Jiancheng, Nanjing, China), respectively. All samples were taken from the third leaf of tobacco.

### UPLC-MS/MS for the determination of salt stress-related substances

Tryptophan and tryptamine were measured as follows: 4.5 g of WT tobacco and transgenic tobacco leaf samples were used for this experiment and transgenic samples from three transgenic strains of 1.5 g each. And the samples were ground thoroughly under liquid nitrogen, then they were transferred into 50 mL centrifuge tubes. 40 ml methanol was added to each tube and then shook for 2 min. An ultrasonic water bath (200 W, 20°C) on ice for 30 minutes to aid and facilitate the extraction of substances such as melatonin. Centrifuge at 4°C, 12000 revolutions per minute (rpm) for 5 min, collect the supernatant, and freeze dry overnight. Re-solve with 2.5 mL of methanol, invert up and down to fully dissolve, and centrifuge at 4°C for 5 min at 12000 rmp to collect the supernatant. The supernatant was filtered through a 0.22 μm filter into a brown bottle for high-performance liquid chromatography-tandem mass spectrometry (UPLC-MS/MS) analysis. Agilent 1290-6495 UPLC-MS/MS (Agilent, Waldbronn, Germany) was used for the experiment. Each sample was tested on a C18 column using the following parameters: an ion source, AJS-ESI +; acquisition mode, MRM mode; dry gas flow rate, 14 L/min; dry gas temperature, 250°C; nitrogen pressure, 30 psi; sheath gas flow rate, 12 L/min; sheath gas temperature, 375°C; capillary voltage, 4,000 V; and nozzle voltage, 500 V. The mobile phases were 0.1% formic acid in water (A) and MeOH (B). The gradient elution program was set as follows: 0–2 min, 10% (B); 2–4 min, 10–90% (B); 4–6 min, 90–98% (B); 6–8 min, 98–10% (B). The flow rate was set at 0.15 mL/min and the injection volume was set at 3.0 µL. serotonin, N-acetylserotonin, and melatonin assays refer to previously published articles (Zheng et al., 2021b). The content of each sample was determined in triplicate.

### Statistical analyses

Relative gene expression values were determined using the 2^−ΔΔCt^ method (Livak and Schmittgen, 2001). Statistical data on melatonin and its precursor content, relative gene expression, and root length of plantlets was organized in Excel 2016 (Microsoft, Redmond, WA, USA). The results were presented as mean values ± SDs. The significant differences between samples were analyzed using a one-way ANOVA in SPSS Statistics 16.0 (SPSS Inc., Chicago, IL, USA) and GraphPad Prism 7.0 (GraphPad Software, La Jolla, USA).

## Result

### Cloning of *MnT5H2* and acquisition of transgenic strains

**Figure 1:**
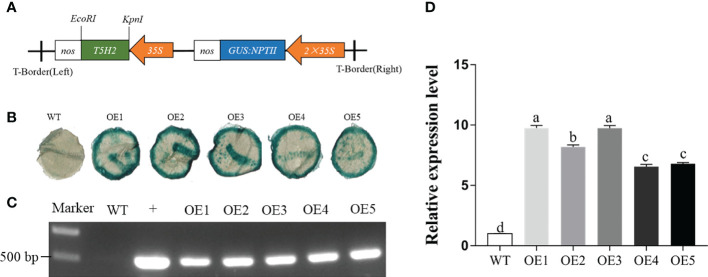
The transfer of *MnT5H2* into tobacco. **(A)** Structure diagram of the pLGNL-*MnT5H2* vectors. *NPTII*: neomycin phosphotransferase II gene. 35S: cauliflower mosaic virus 35S promoter. **(B)** Histochemical GUS staining of transgenic lines. **(C)** Kanamycin resistance gene *NPTII* positive identification of genome (+): pLGNL-*MnT5H2* vector. **(D)** Quantitative real-time PCR analysis. WT, The wild tobacco; OE, The overexpressed transgenic plants. Data are means ± SDs (n=3). Different letters above the bars indicate the significant difference of different lines at *P*<0.05.

To generate transgenic tobacco plants overexpressing *MnT5H*, we employed the pLGNL vector. Given that *T5H2* has the highest expression in *Morus notabilis*, *MnT5H2* was obtained by amplifying the cDNA as a template with specific primers. We successfully constructed the pLGNL-*MnT5H2* overexpression vector and transferred it into *Agrobacterium* tumefaciens receptor cells (**Figure 1A**). Positive *Agrobacterium* strains were transformed into tobacco and five independently overexpressed transgenic line (OE) plants were obtained. For further identification of positive transgenic lines, we performed GUS staining and genomic PCR identification of transgenic lines. In the GUS staining solution, all five *MnT5H2* transgenic lines were identified as positive, showing a blue-green color (**Figure 1B**). When the *NPTII* sequence was amplified from the genome, the bands were amplified in the genomes of the five *MnT5H2* transgenic lines, and no target gene-specific bands were amplified in WT tobacco (**Figure 1C**). Among these transgenic tobaccos, OE1 had the highest expression and OE4 had the lowest expression. Analysis of gene expression showed no significant difference between OE1 and OE3 and no significant difference in the expression of OE4 and OE5. Given this, in the subsequent experiments, we selected OE1, OE2, and OE5 transgenic lines with significantly different expressions as the experimental materials (**Figure 1D**).

### Overexpression of *MnT5H2* increases melatonin and serotonin content in transgenic tobacco

**Figure 2:**
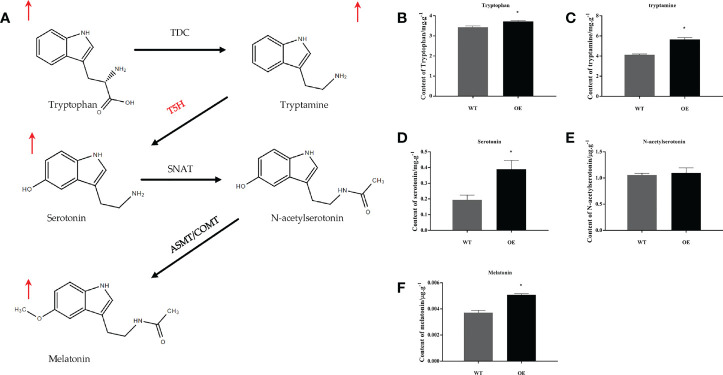
The precursors of melatonin and melatonin content in the tobacco leaf extract. **(A)** The biosynthetic pathway of melatonin in plants; **(B)** Tryptophan; **(C)** tryptamine; **(D)** Serotonin; **(E)** N-acetylserotonin; **(F)** Melatonin. Red arrows indicate a significant increase in content WT: The wild-type tobacco; OE: The overexpressed transgenic tobacco. Data are means ± SDs (n=3). The * above the bars indicates the significant difference of different lines at *P*<0.05.

UPLC-MS/MS analysis of substances on the melatonin synthesis pathway in *MnT5H2* transgenic tobacco and WT tobacco showed that L-tryptophan, tryptamine, serotonin, N-acetylserotonin, and melatonin were detected. Except for N-acetylserotonin, the levels of substances in the melatonin synthesis pathway were significantly increased (**Figure 2A, E**). Especially serotonin, the content increased by about 1-fold (**Figure 2D**). A significant increase in the level of serotonin, the product of *MnT5H2*, is reasonable and it may also account for the increase in the level of subsequent products. Levels of tryptophan and tryptamine also increased significantly, though this trend was not very tremendously (**Figures 2B, C**).

### Overexpression of *MnT5H2* improves salt tolerance in transgenic tobacco

**Figure 3:**
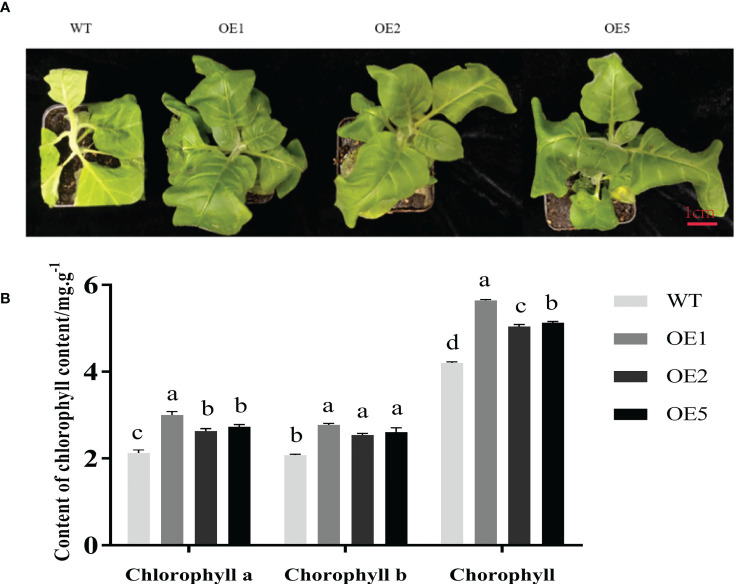
Growth and chlorophyll content of tobacco under salt stress. **(A)** The phenotype of tobacco after 10 days of salt stress. **(B)** Chlorophyll content of tobacco after 10 days of salt stress. Error bars on each column indicate SDs (n=3). The letters above the bars indicate the significant difference of different lines at *P*<0.05.

After salt stress treatment, there were significant differences in leaf phenotypes between the three transgenic lines and WT tobacco (**Figure 3A**). Mainly manifested in the degree of leaf yellowing, plant growth height, and the degree of leaf wilting. Phenotypically, the transgenic plants showed weaker leaf inhibition than WT tobacco, indicating that the transgenic strains had better tolerance to salt stress than the WT. Chlorophyll content reflects the health of tobacco. The chlorophyll, chlorophyll a, and chlorophyll b contents of transgenic plants were significantly higher than those of WT (**Figure 3B**). These results show that *MnT5H2*-overexpression tobacco had better salt tolerance.

### Changes in the content of substances related to the melatonin biosynthesis pathway

**Figure 4:**
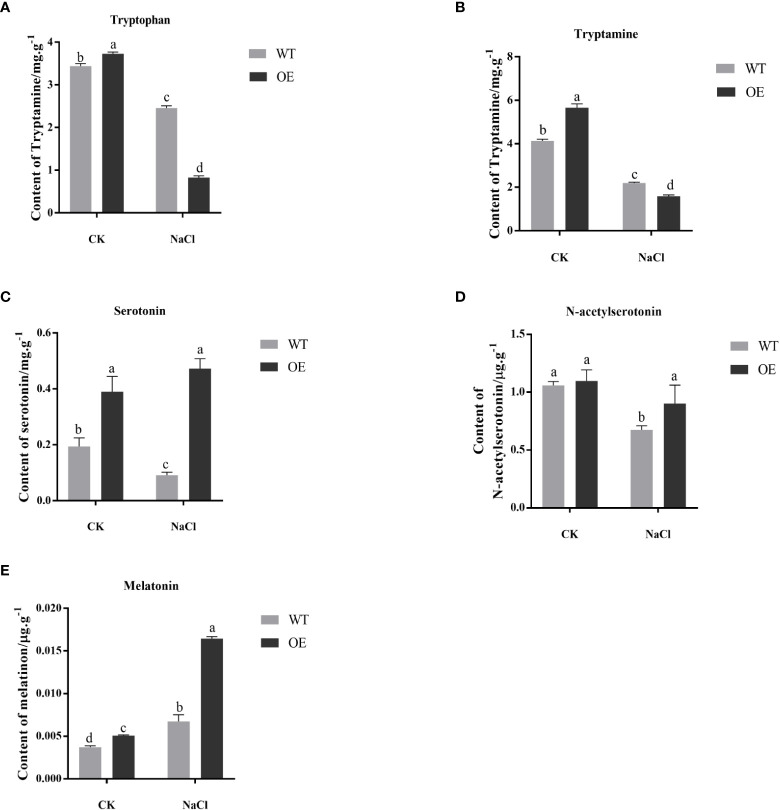
Comparative analysis of the content of substances related to melatonin synthesis in tobacco **(A)** L-tryptophan; **(B)** Tryptamine; **(C)** Serotonin; **(D)** N-acetylserotonin; **(E)** Melatonin. CK, control, without NaCl stress. WT, The wild tobacco; OE, The overexpressed transgenic plants. Error bars on each column indicate SDs (n=3). The letter above the bars indicates the significant difference of different lines at *P*<0.05.

After salt stress, each substance related to the melatonin synthesis pathway was extracted from the transgenic and WT tobacco, and further identified and detected by UPLC-MS/MS. The NaCl stress significantly decreased the tryptophan content of transgenic and WT tobacco. And under salt stress, the tryptophan content of transgenic tobacco was significantly lower than that of WT (**Figure 4A**). Tryptamine has the same trend as tryptophan (**Figure 4B**). Salt stress significantly reduced the serotonin content of WT tobacco but had almost no effect on transgenic tobacco. And serotonin content of transgenic tobacco was significantly higher than that of WT under salt stress (**Figure 4C**). N-acetylserotonin has the same trend as Serotonin (**Figure 4D**). In both WT and transgenic, melatonin content was significantly elevated after salt treatment. And under salt stress, the melatonin content of transgenic tobacco was significantly higher than that of WT (**Figure 4E**). These results indicated that, under salt stress, *MnT5H2* actively promoted the conversion of melatonin synthesis precursors, especially tryptophan and tryptamine, to the latter substances and eventually led to an increase in melatonin content and increased resistance to stress.

### Comparison of physiological indicators of tobacco under NaCl stress

**Figure 5:**
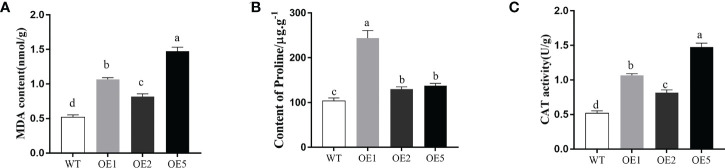
Determination of physiological and biochemical indexes of transgenic tobacco after salt treatment. **(A)** The content of malondialdehyde (MDA). **(B)** The content of proline. **(C)** The activities of catalase. Error bars on each column indicate SDs (n=3). The letters above the bars indicate the significant difference of different lines at *P*<0.05.

The proline and CAT play important role in plant adaption to abiotic stress. The MDA content, proline content, and CAT enzyme activity of the leaves of *MnT5H2*-overexpressing tobacco and WT tobacco were examined after treatment with 300 mM NaCl for 10 d. MDA content in transgenic was significantly lower than in WT tobacco (**Figure 5A**). The CAT enzyme activity and Pro content were significantly greater in the transgenic strain than in WT (**Figures 5B, C**). These results suggest that overexpression of the *MnT5H2* in tobacco improves osmoregulation and scavenging of reactive oxygen species in tobacco.

### Comparison of expression of stress-responsive genes in tobacco under NaCl stress

**Figure 6:**
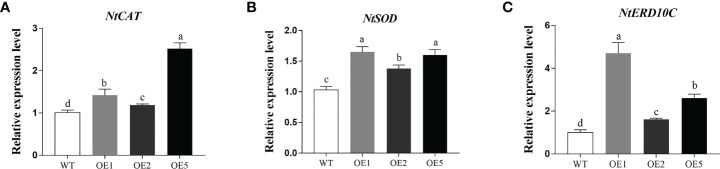
Quantification of stress-related genes in *MnT5H2* transgenic tobacco after Salt Stress. **(A)** The Relative expression level of *NtSOD*. **(B)** The Relative expression level of *NtCAT*. **(C)** The Relative expression level of *NtRED10C*. Error bars on each column indicate SDs (n=3). The letters above the bars indicate the significant difference of different lines at *P*<0.05.

Compared to WT, the expression of antioxidant enzyme genes superoxide dismutase (*NtSOD*) and *NtCAT* were significantly increased in the transgenic tobacco after salt stress (**Figures 6A, B**). The expression of early response to drought 10 (*NtERD10C*) gene, which regulates osmotic pressure homeostasis, was also significantly increased (**Figure 6C**). These results showed that overexpression of *MnT5H2* induces up-regulated expression of *NtSOD*, *NtCAT*, and *NtERD10C* genes, which can improve osmoregulation and scavenging of reactive oxygen species in tobacco.

### Transgenic tobacco seedlings grow faster than WT under salt stress

**Figure 7:**
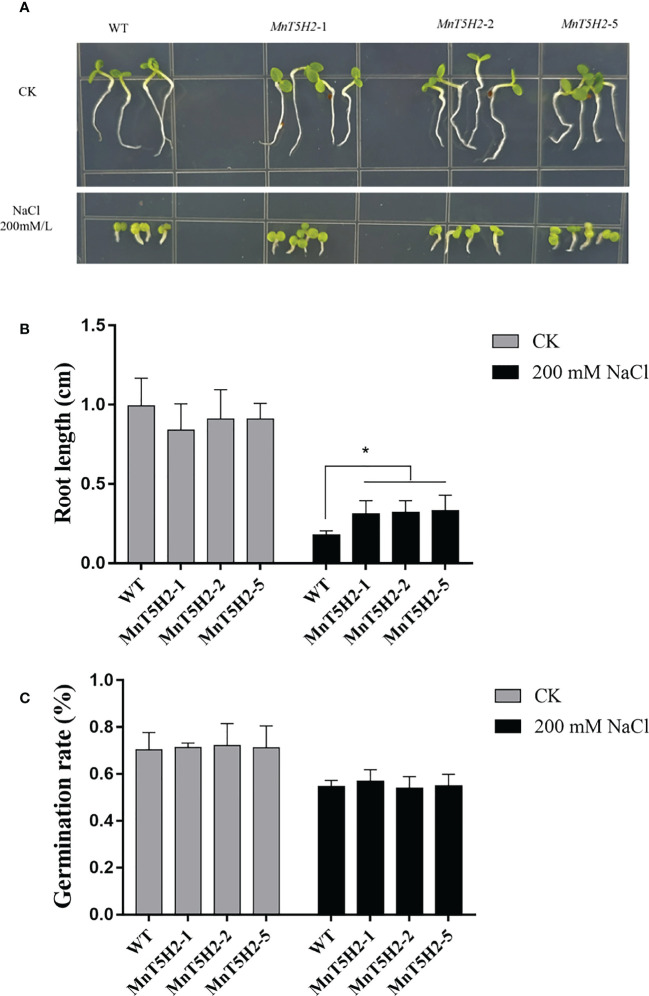
Germination and growth of transgenic tobacco in the salt-containing medium. **(A)** The phenotype of tobacco after 12 days of germination and growth in salt-containing 1/2 MS medium. **(B)** Root length statistics. Error bars on each column indicate SDs (n=12). **(C)** Germination rate statistics. Error bars on each column indicate SDs (n=3). The * above the bars indicates the significant difference of different lines at *P*<0.05.

After 12 days of germination and growth in a 1/2 MS medium containing NaCl (200 mM), it was found that the transgenic tobacco seedlings were better adapted to the high salt environment (**Figure 7A**). After 12 days of germination of the seeds of transgenic strains and WT in NaCl-containing medium, the root length of transgenic tobacco (*MnT5H2*-1 0.31 ± 0.08 cm, *MnT5H2*-2 0.32 ± 0.07 cm, *MnT5H2*-5 0.33 ± 0.09 cm) was significantly longer than that of WT (0.17 ± 0.02 cm). And there was no significant difference in the root length of tobacco grown in NaCl-free medium (**Figure 7B**). However, there was no significant difference in germination rate between WT and transgenic (**Figures 7C**, **S1**). These results demonstrate that *MnT5H2* did not contribute to tobacco seed germination under 200 mM NaCl stress, but influenced seedling resistance to salt stress.

## Discussion

The transgenic tobacco treated with salt stress had a more significant growth advantage than the WT under 300 mM salt stress. In multiple replicates, both WT and transgenic tobacco exhibited significant leaf yellowing and growth retardation, but transgenic tobacco performed significantly better. Similar outcomes were observed in *VvSNAT-*overexpression *Arabidopsis* and *NtCOMT-*overexpression tobacco (Wu et al., 2021; Yao et al., 2022). Extensive studies have shown that melatonin plays a vital role in increasing salt tolerance in various plant species (Jalili et al., 2022; Song et al., 2022b). The exogenous addition of melatonin increases the resistance of plants to salt stress, and we consider that this increase in resistance in *MnT5H2* transgenic plants is most likely related to their increased melatonin content. Unfortunately, this complex mechanism is not addressed in our work. Melatonin has similar physiological functions to auxin, and its specific mechanism in plants is complex and needs further study (Hernandez-Ruiz et al., 2005; Friml et al., 2022).

Extraction of melatonin synthesis pathway-related substances from transgenic tobacco and WT tobacco after salt stress, and further validation of the content of each substance by UPLC-MS/MS. The levels of tryptophan and tryptamine, precursors of serotonin synthesis, were significantly elevated. The same was observed under exogenous addition, which may be related to the positive feedback regulation of melatonin (Song et al., 2022a).

Among these substances, we extra focus on serotonin the products of *MnT5H2*, and melatonin. Compared to WT, serotonin and melatonin were significantly increased in transgenic tobacco. Interestingly, the amount of serotonin in it rose to about 0.2 mg/g, a 2-fold increase. This trend was further enlarged under salt stress. Serotonin has been confirmed in almost all plant families, where it plays important role in plant growth and development (Erland et al., 2016). It also an analogue of IAA, is involved in the regulation of lateral root growth in *Arabidopsis* (Pelagio-Flores et al., 2011; Wan et al., 2018). Some studies have reported that overexpression of *T5H* will lead to increased serotonin content and decreased melatonin content in transgenic plants (Park et al., 2012; Park et al., 2013a). In this study, we found both melatonin and serotonin increased in *MnT5H2* transgenic plants. This provides new insights into the relationship between serotonin and melatonin. Furthermore, the increase in serotonin in transgenic tobacco may also be positively related to its increased salt tolerance.

**Figure 8:**
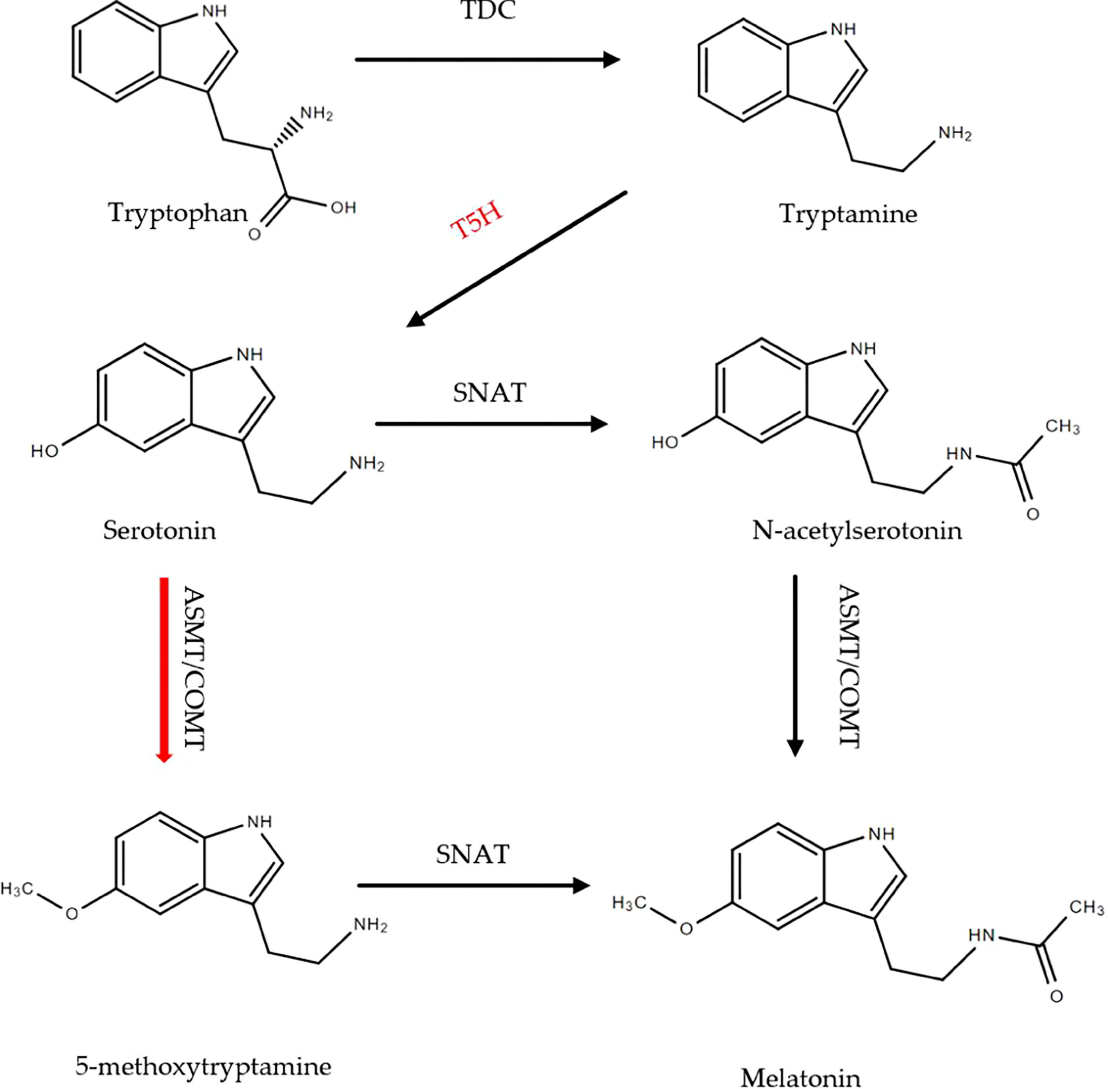
The pathway of melatonin synthesis in tobacco. Red arrows indicate the preference of chemical reactions.

The classic melatonin synthetic pathway has been described in this article (**Figure 2A**). However, in this study, it was found that *MnT5H2* overexpression resulted in increased melatonin synthesis, but in the absence of N-acetylserotonin changes. This indicates that there are other pathways for melatonin synthesis in tobacco. Previous studies have shown that the sequence of roles of SNAT and ASMT in the melatonin synthesis pathway can be altered (Lee et al., 2014; Tan et al., 2016). It has also been reported that these two enzymes have different substrate selection preferences at different temperatures and that ASMT is the rate-limiting enzyme for melatonin synthesis (Reiter et al., 2007; Kang et al., 2013). Our result suggests that there may be a more preferential synthetic pathway for tobacco melatonin that differs from the classical pathway (**Figure 8**). The significance of this different pathway synthesis mechanism needs to be further investigated.

Referring to our previous study, we also counted tobacco seeds germinating and growing at specific salt concentrations (Liu et al., 2018). Interestingly, transgenic tobacco T1 seeds did not exhibit higher germination rates, but after germination transgenic tobacco seedlings grew significantly better than WT under salt stress, as evidenced by the fact that transgenic tobacco seedlings could grow slowly in salt-stressed media, but WT seedlings almost stopped growing (**Figure 7**). Exogenous melatonin has been reported to promote seedling growth and to promote seed germination (Park and Back, 2012; Nabaei and Amooaghaie, 2019; Li et al., 2022; Wang J. et al., 2022). This study found that the promotion of seedling growth by *MnT5H2* may be related to the increased synthesis of endogenous melatonin. However, the absence of effect on seed germination may be related to the specific localization of other enzymes, such as SNAT whose main activity is concentrated in chloroplasts (Byeon et al., 2014).

Consuming melatonin from plant-based foods or supplementing with melatonin may promote health by virtue of its many properties (Iriti et al., 2010; Favero et al., 2017). Medicinal herbs are often rich in melatonin, while many vegetables and fruits are low in melatonin and vary greatly between species (Cheng G. et al., 2021). Overexpression of highly efficient melatonin synthesis genes *MnT5H* resulted in a dramatic increase in melatonin levels in tobacco. This provides useful ideas and references for improving melatonin accumulation in vegetables.

When plants are stressed, the dynamic balance of ROS production and elimination in plant cells is disrupted. Excess ROS can disrupt the cell membrane system and negatively affect the growth of plants (Pardo-Hernandez et al., 2021). The indicators of ROS were examined in tobacco overexpression *MnT5H2* after salt stress, and it was found that CAT enzyme and POD enzyme activities involved in antioxidant were higher in transgenic tobacco. And the content of MDA was decreased, the content of proline was increased. The result indicated that *MnT5H2* enhances salt resistance in tobacco through antioxidant properties and ion homeostasis. The conclusion was further supported by expression analysis by real-time PCR of *NtSOD*, *NtCAT*, and *NtERD10C* (Liu et al., 2013). Under salt stress, the expression of those genes in transgenic tobacco was significantly higher than that in WT tobacco. *MnT5H2* enhances plant tolerance to saline environments by up-regulating the expression of related genes, enhancing the activity of antioxidant enzymes, and increasing the content of protective organisms and antioxidant-related substances.

In this study, after overexpression of the *MnT5H2* gene, melatonin content was significantly increased in transgenic tobacco, and melatonin level was still significantly higher in transgenic tobacco than in WT tobacco under salt stress treatment. As melatonin can act as a strong antioxidant in the plant body to eliminate the reactive oxygen species stress caused by the adverse environment, the physiological and biochemical indicators of transgenic tobacco showed a more positive trend than WT tobacco. In contrast to the results of previous studies (Park et al., 2012; Park et al., 2013a), we believe that the improved salt tolerance of transgenic tobacco is due to the overexpression of *MnT5H2*, which ultimately increases the amount of melatonin synthesis in the tobacco and the increased level of melatonin enhances the salt tolerance of transgenic tobacco. This reveals that the *MnT5H* gene is of great value in both the vegetable industry and horticulture.

## Data availability statement

The original contributions presented in the study are included in the article/**Supplementary Material**. Further inquiries can be directed to the corresponding author.

## Author contributions

BZ, SZ, and AZ conceived and designed the experiments. BZ, MZ, and XC performed the experiments. BZ, WF, ZX, and AZ wrote the article. All authors approved the final manuscript. All authors contributed to the article and approved the submitted version.

## Funding

This research was funded by the China Agriculture Research System of MOF and MARA, grant number CARS-18-ZJ0201.

## Conflict of interest

The authors declare that the research was conducted in the absence of any commercial or financial relationships that could be construed as a potential conflict of interest.

## Publisher’s note

All claims expressed in this article are solely those of the authors and do not necessarily represent those of their affiliated organizations, or those of the publisher, the editors and the reviewers. Any product that may be evaluated in this article, or claim that may be made by its manufacturer, is not guaranteed or endorsed by the publisher.
