# Supplementary material for: Ectopic overexpression of mulberry MnT5H2 enhances melatonin production and salt tolerance in tobacco
Source: Front Plant Sci. 2022 Nov 25;13:1061141. doi: 10.3389/fpls.2022.1061141 (PMC9733638; doi:10.3389/fpls.2022.1061141)
Supplement: Supplementary file 1 [file DataSheet_1.docx]

Supplementary Material

# Supplementary Figures and Tables

## Supplementary Table 1.

Primer sequences used in this study.

| **Name** | **Primers** |
| --- | --- |
| *MnT5H2*-F | GGGGTACCATGGCTCTCCTTCAGTGGTT ^1^ |
| *MnT5H2*-R | CGGAATTCTTATGGAAAGCGTGGCTTGG |
| *MnT5H*-F | CCCGGCACAACCAATCTAAG |
| *MnT5H*-R | AAAGCGTGCACAAACTCCAT |
| *NtActin*-F | TCACAGAAGCTCCTCCTAATCCA |
| *NtActin*-R | GAGGGAAAGAACAGCCTGAATG |
| *NtCAT*-F | AGGTACCGCTCATTCACACC |
| *NtCAT*-R | AAGCAAGCTTTTGACCCAGA |
| *NtSOD*-F | AGCTACATGACGCCATTTCC |
| *NtSOD*-R | CCCTGTAAAGCAGCACCTTC |
| *NtERD10C-F* | GGAAGAAGAGAAGGCGGGTGA |
| *NtERD10C-R* | GGTCTTTGAGTGATATCCTGGTA |

^1^Sequence underline is restriction site, before restriction site there are 2 protection bases.

## Supplementary Figure1.

##
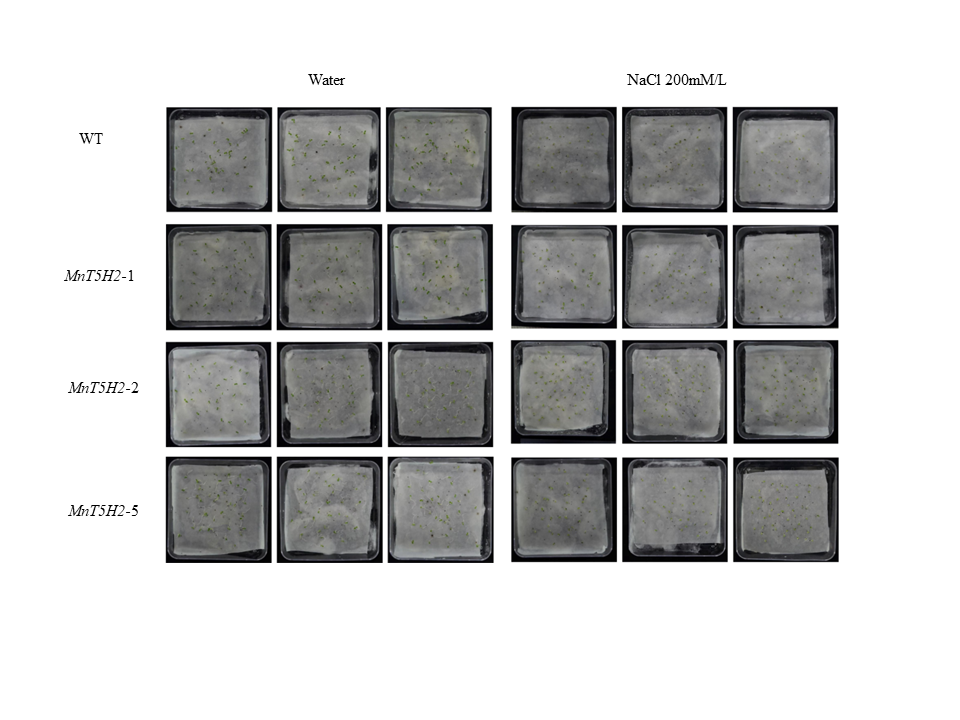


**Figure S1**. Germination of transgenic tobacco and wild type under salt stress.
